# Predicting estimated glomerular filtration rate after partial and radical nephrectomy based on split renal function measured by radionuclide: a large-scale retrospective study

**DOI:** 10.1007/s00345-023-04686-4

**Published:** 2023-10-31

**Authors:** Wanxiang Zheng, Guangdong Hou, Dongen Ju, Fei Yan, Kepu Liu, Zhiping Niu, Luguang Huang, Zibao Xing, Lingchen Kong, Pengfei Liu, Geng Zhang, Di Wei, Jianlin Yuan

**Affiliations:** 1 Department of Urology, Xijing Hospital, Fourth Military Medical University, Xi’an, China; 2 School of Pharmacy, Health Science Center, Xi’an Jiaotong University, Xi’an, China; 3 Information Center, Xijing Hospital, Fourth Military Medical University, Xi’an, China; 4 Department of Urology, The 73rd Army Group Hospital, Xiamen, China; 5 Air Force Hospital of Western Theater Command, PLA, Chengdu, China

**Keywords:** Glomerular filtration rate, Partial nephrectomy, Radical nephrectomy, Renal function, Tc-99 m DTPA

## Abstract

**Purpose:**

The purpose of this study was to develop predictive models for postoperative estimated glomerular filtration rate (eGFR) based on the split glomerular filtration rate measured by radionuclide (rGFR), as choosing radical nephrectomy (RN) or partial nephrectomy (PN) for complex renal masses requires accurate prediction of postoperative eGFR.

**Methods:**

Patients who underwent RN or PN for a single renal mass at Xijing Hospital between 2008 and 2022 were retrospectively included. Preoperative split rGFR was evaluated using technetium-99 m-diethylenetriaminepentaacetic acid (Tc-99 m DTPA) renal dynamic imaging, and the postoperative short-term (< 7 days) and long-term (3 months to 5 years) eGFRs were assessed. Linear mixed-effect models were used to predict eGFRs, with marginal *R*^*2*^ reflecting predictive ability.

**Results:**

After excluding patients with missing follow-up eGFRs, the data of 2251 (RN: 1286, PN: 965) and 2447 (RN: 1417, PN: 1030) patients were respectively included in the long-term and short-term models. Two models were established to predict long-term eGFRs after RN (marginal *R*^2^ = 0.554) and PN (marginal *R*^2^ = 0.630), respectively. Two other models were established to predict short-term eGFRs after RN (marginal *R*^2^ = 0.692) and PN (marginal *R*^2^ = 0.656), respectively. In terms of long-term eGFRs, laparoscopic and robotic surgery were superior to open surgery in both PN and RN.

**Conclusions:**

We developed novel tools for predicting short-term and long-term eGFRs after RN and PN based on split rGFR that can help in preoperative decision-making.

**Supplementary Information:**

The online version contains supplementary material available at 10.1007/s00345-023-04686-4.

## Introduction

Radical nephrectomy (RN) and partial nephrectomy (PN) are both the most effective ways to treat localized renal masses. In addition to oncological outputs, clinicians also prioritize postoperative renal function (RF), with estimated glomerular filtration rate (eGFR) being the key indicator representing total RF. PN can better preserve the eGFR than RN [1]. However, the beneficial impact of PN does not seem to translate into survival benefits unless the preoperative eGFR is already significantly impaired [2–4]. Therefore, patients with high tumor complexity, no preexisting chronic kidney disease, or proteinuria are recommended to undergo RN rather than PN if the expected eGFR is greater than 45 ml/min/1.73 m^2^ after RN [5]. In these cases, the downside of RN appears to be inconsequential [6], and PN is associated with an increased risk of perioperative morbidity and compromised oncologic outcomes. Moreover, clinicians should consider referring patients to nephrology if the expected eGFR is not satisfactory after intervention [5]. All of these aspects require accurate predictions of postoperative eGFR.

Unfortunately, there are few models to predict postoperative eGFR. Information about preoperative split RF is common lacking [7–10], which results in limited ability in predicting eGFR after RN [11]. Other studies estimated split RF from images using parenchymal volume analysis [11–15]. These methods require volume calculating software [13] and training, and are not suitable for routine applications. Furthermore, these studies were limited by small sample sizes. Numerous medical institutions routinely use technetium-99 m-diethylenetriaminepentaacetic acid (Tc-99 m DTPA) renal dynamic imaging (RDI) to assess preoperative split glomerular filtration rate measured by radionuclide (rGFR). However, its role in predicting postoperative eGFR is still far from clear, and its application is based on superficial clinical experience.

Thus, this study aimed to develop preoperative tools for predicting postoperative eGFR based on split rGFR. Separate models predicting short-term and long-term eGFRs were constructed to cover almost all periods after surgery.

## Methods

### Study design and participants

In this retrospective cohort study, data on patients with a single, unilateral renal mass who underwent RN or PN at Xijing Hospital from August 2008 to March 2022 were collected. Patients with preoperative renal failure (eGFR < 15 ml/min/1.73 m^2^) were excluded.

We collected these variables as potential predictors of postoperative eGFR based on previous research, including age, gender, body mass index (BMI), tumor size, preoperative eGFR, rGFR of the operative side, rGFR of the healthy side, serum cystatin C, diabetes, hypertension, surgical approach (open, laparoscopic, or robot-assisted laparoscopic surgery), and time from surgery (TFS).

### Follow-up and study outcomes

The single-kidney glomerular filtration rate (GFR) was routinely calculated by Tc-99 m DTPA RDI using the Gate’s method before surgery [16]. During hospitalization, serum creatinine was rechecked within 7 days postoperatively. The planned follow-up included serum creatinine measurements at 3 months, 6 months, and 1 year postoperatively and then annually thereafter until 5 years.

The eGFRs were calculated according to the Chronic Kidney Disease Epidemiology Collaboration formula [17]. The long-term models predicted the eGFRs at 3 months, 6 months, and 1–5 years postoperatively. The short-term models predicted the eGFRs at 1–7 days postoperatively.

### Statistical analysis

The association between preoperative characteristics and postoperative eGFRs was evaluated using linear mixed-effect models with random patient-specific intercepts. Moreover, we introduced a temporal autocorrelation structure for each model [18].

Multivariable models were developed using backward selection of all the preoperative factors and their two-way interactions with TFS, with the significance level set to 0.01. *P*-values were provided using Satterthwaite's degrees of freedom method via the R package *lmerTest*. Main effects were included whenever the interaction with TFS was retained in model selection [7].

The correlations between the preoperative variables were investigated using the Spearman correlation test. In the process of performing the Spearman correlation test and establishing multivariable models, patients without complete data for all preoperative features were excluded.

Marginal and conditional *R*^2^ were used to summarize the predictive ability of the models [19], with the value of 0 representing no predictive ability and 1.0 representing perfect predictive ability. The marginal *R*^2^ helped describe the ability of preoperative characteristics to predict eGFR in future patients. The conditional *R*^2^ also included between- and within-patient variability and can be considered as the total variation that can be explained by the models.

Statistical analyses were performed using SPSS version 26.0 (SPSS Inc., Chicago, IL, USA) for Windows and R version 4.2.2 (R Foundation for Statistical Computing, Vienna, Austria). The measurement data with a nonnormal distribution are presented as the median (interquartile range, IQR).

## Results

### Patient characteristics

The study flowchart is shown in Supplementary Fig. 1.

2539 patients (RN: 1417, PN: 1122) with complete preoperative information were enrolled in the analyze about the correlations among preoperative predictors.

2251 patients [RN: 1286 (786 males, 500 females), PN: 965 (671 males, 294 females)] with at least one long-term (> 3 months) follow-up eGFR were enrolled in the development of long-term model (Table 1). The age was 55 (48–64) years for RN patients, and was 54 (46–63) years for PN patients. The tumor size was 5 (3.9–6.8) cm for RN patients, and was 3.4 (2.5–4.2) cm for PN patients. The preoperative eGFR was 75.5 (65.3–89.8) ml/min/1.73 m^2^ for RN patient, and was 79.6 (68.2–96.3) ml/min/1.73 m^2^ for PN patients. The follow-up time after RN and PN was both 24 (6–48) months. In the long-term follow-up of RN patients, no cases had renal failure. However, in the long-term follow-up of PN patients, renal failure was observed in 2 patients, with the initial cases occurring at 3 years and 4 years, respectively.

**Table 1:** Preoperative cohort characteristics for the long-term model and short-term model

Long-term model			Short-term model		
Feature	Median (quartile) / N (%)		Feature	Median (quartile) / N (%)	
	RN (N = 1286)	PN (N = 965)		RN (N = 1417)	PN (N = 1030)
Age (year)	55 (48–64)	54 (46–63)	Age (year)	57 (49–65)	56 (48–65)
BMI (kg/m^2^; N = 1199:914)	24.3 (22.2–26.5)	25.1 (22.8–27.4)	BMI (kg/m^2^; N = 1297:982)	24.2 (22–26.3)	25 (22.8–27.6)
Tumor size (cm; N = 1284:965)	5 (3.9–6.8)	3.4 (2.5–4.2)	Tumor size (cm; N = 1414:1030)	5.1 (4–7)	3.3 (2.5–4.2)
Preoperative eGFR (ml/min/1.73 m^2^; N = 1275:961)	75.5 (65.3–89.8)	79.6 (68.2–96.3)	Preoperative eGFR (ml/min/1.73 m^2^; N = 1403:1023)	75.9 (65–89.8)	80.4 (68–96.9)
RGFR of the operative side (ml/min/1.73 m^2^; N = 1136:851)	39.8 (31.7–48.2)	45.5 (37.7–53.7)	RGFR of the operative side (ml/min/1.73 m^2^; N = 1230:907)	38.4 (29.8–46.7)	44.7 (36.9–53.4)
RGFR of the healthy side (ml/min/1.73 m^2^; N = 1136:851)	46.1 (40–54.7)	45.6 (38–54.3)	RGFR of the healthy side (ml/min/1.73 m^2^; N = 1230:907)	45 (39.2–53.8)	45.2 (37.7–53.2)
Serum cystatin C (mg/L; N = 1100:938)	0.97 (0.85–1.09)	0.91 (0.79–1.04)	Serum cystatin C (mg/L; N = 1143:1002)	0.98 (0.86–1.12)	0.92 (0.8–1.05)
Female sex	500 (39)	294 (31)	Female sex	521 (37)	306 (30)
Diabetes (N = 1272:955)	123 (10)	133 (14)	Diabetes (N = 1400:1018)	144 (10)	154 (15)
Hypertension (N = 1281:961)	359 (28)	307 (32)	Hypertension (N = 1410:1027)	396 (28)	350 (34)
Surgical method			Surgical method		
Open surgery	394 (31)	131 (13)	Open surgery	514 (36)	125 (12)
Laparoscopic surgery	841 (65)	450 (47)	Laparoscopic surgery	841 (59)	510 (50)
Robotic surgery	51 (4)	384 (40)	Robotic surgery	62 (5)	395 (38)

*BMI* body mass index; *eGFR* estimated glomerular filtration rate; *PN* partial nephrectomy; *rGFR* glomerular filtration rate measured by radionuclide; *RN* radical nephrectomy

2447 patients (RN: 1417, PN: 1030) with at least one short-term (< 7 days) follow-up eGFR were enrolled in the development of short-term model (Table 1). The short-term follow-up time after RN was 3 (1–5) days. The short-term follow-up time after PN was 2 (1–4) days. Within 7 days after RN, no cases had renal failure. However, within 7 days after PN, 2 patients experienced renal failure, with the first occurrence observed both at 2 days.

### The correlation analysis of preoperative predictors

The correlations among preoperative predictors were shown in the heat plot (Supplementary Fig. 2). Strong positive correlations were observed between the rGFR of the operative side and rGFR of the healthy side (r = 0.531, *P* < 0.001) and between age and serum cystatin C (r = 0.404, *P* < 0.001). Additionally, strong negative correlations were observed between the rGFR of the operative side and serum cystatin C (r =  − 0.450, *P* < 0.001), between the rGFR of the healthy side and serum cystatin C (r =  − 0.420, *P* < 0.001), and between age and preoperative eGFR (r =  − 0.411, *P* < 0.001).

### Predicting long-term eGFR beyond 3 months postoperative

A multivariable model was constructed to predict the eGFR beyond 3 months after RN (Table 2, Supplementary Table 1): postoperative eGFR ~ Intercept + age + preoperative eGFR + rGFR of the operative side + rGFR of the healthy side + serum cystatin C + diabetes + surgical method + TFS + preoperative eGFR × TFS + rGFR of the healthy side × TFS + serum cystatin C × TFS + surgical method × TFS. Older age, lower preoperative eGFR, higher rGFR of the operative side, lower rGFR of the healthy side, higher serum cystatin C, diabetes, open surgical approach, and shorter TFS were associated with worse long-term eGFR. Moreover, patients with higher preoperative eGFR, lower rGFR of the healthy side, higher serum cystatin C, and open surgical approach had less recovery of eGFR per unit time. The marginal and conditional *R*^2^ values were 0.554 and 0.663, respectively.

**Table 2:** Features of the multivariable models to predict long-term postoperative eGFR

Feature	Estimate	SE	*P* value
RN			
Intercept	4.37	4.27	0.31
Age (year)	– 0.105	0.0329	0.001
Preoperative eGFR (ml/min/1.73 m^2^)	0.674	0.0259	< 0.001
RGFR of the operative side (ml/min/1.73 m^2^)	– 0.152	0.0278	< 0.001
RGFR of the healthy side (ml/min/1.73 m^2^)	0.204	0.0288	< 0.001
Serum cystatin C (mg/L)	0.914	2.30	0.69
Diabetes	– 3.57	1.12	0.002
Surgical method			
Open surgery	Reference		
Laparoscopic surgery	0.480	0.975	0.62
Robotic surgery	3.96	1.93	0.04
Time from surgery (month)	0.930	0.137	< 0.001
Preoperative eGFR × time from surgery (month)	– 0.00686	0.00101	< 0.001
RGFR of the healthy side × time from surgery (month)	0.00443	0.00107	< 0.001
Serum cystatin C × time from surgery (month)	– 0.452	0.0826	< 0.001
Surgical method × time from surgery (month)			
Open surgery	Reference		
Laparoscopic surgery	0.105	0.0316	0.001
Robotic surgery	0.0426	0.0719	0.55
PN			
Intercept	15.3	4.91	0.002
Age (year)	– 0.103	0.0463	0.03
Preoperative eGFR (ml/min/1.73 m^2^)	0.864	0.0298	< 0.001
Serum cystatin C (mg/L)	– 6.62	2.64	0.01
Surgical method			
Open surgery	Reference		
Laparoscopic surgery	2.23	1.63	0.17
Robotic surgery	2.42	1.64	0.14
Time from surgery (month)	1.78	0.189	< 0.001
Age × time from surgery (month)	– 0.00672	0.00177	< 0.001
Preoperative eGFR × time from surgery (month)	– 0.0102	0.00128	< 0.001
Serum cystatin C × time from surgery (month)	– 0.556	0.0973	< 0.001
Surgical method × time from surgery (month)			
Open surgery	Reference		
Laparoscopic surgery	0.183	0.0527	< 0.001
Robotic surgery	0.163	0.0531	0.002

*BMI* body mass index; *eGFR* estimated glomerular filtration rate; *PN* partial nephrectomy; *rGFR* glomerular filtration rate measured by radionuclide; *RN* radical nephrectomy

Moreover, a prediction model for the eGFR beyond 3 months after PN was constructed (Table 2, Supplementary Table 2). Older age, lower preoperative eGFR, higher serum cystatin C, open surgical approach, and shorter TFS were associated with worse long-term eGFR. Additionally, patients with older age, higher preoperative eGFR, higher serum cystatin C and open surgical approach had less recovery of eGFR per unit time. The marginal and conditional *R*^2^ values were 0.630 and 0.708, respectively.

### Predicting short-term eGFR within 7 days postoperative

A multivariable model was designed to predict the eGFR within 7 days after RN (Table 3, Supplementary Table 3). Older age, higher BMI, lower preoperative eGFR, higher rGFR of the operative side, lower rGFR of the healthy side and longer TFS indicated worse short-term eGFR. Additionally, patients with higher rGFR of the healthy side had more recovery of eGFR per unit time. The marginal and conditional *R*^2^ values were 0.692 and 0.885, respectively.

**Table 3:** Features of the multivariable models to predict short-term postoperative eGFR

Feature	Estimate	SE	*P* value
RN			
Intercept	28.7	3.47	< 0.001
Age (year)	– 0.145	0.0255	< 0.001
BMI (kg/m^2^)	– 0.489	0.0813	< 0.001
Preoperative eGFR (ml/min/1.73 m^2^)	0.536	0.0174	< 0.001
RGFR of the operative side (ml/min/1.73 m^2^)	– 0.254	0.0218	< 0.001
RGFR of the healthy side (ml/min/1.73 m^2^)	0.263	0.0257	< 0.001
Time from surgery (day)	– 0.784	0.329	0.02
RGFR of the healthy side × time from surgery (day)	0.0331	0.00670	< 0.001
PN			
Intercept	33.9	4.26	< 0.001
Age (year)	– 0.201	0.0387	< 0.001
Tumor size (cm)	– 1.39	0.283	< 0.001
Preoperative eGFR (ml/min/1.73 m^2^)	0.723	0.0252	< 0.001
Serum cystatin C (mg/L)	– 9.15	2.13	< 0.001
Time from surgery (day)	1.58	0.148	< 0.001

*BMI* body mass index; *eGFR* estimated glomerular filtration rate; *PN* partial nephrectomy; *rGFR* glomerular filtration rate measured by radionuclide; *RN* radical nephrectomy

Moreover, a model was constructed to predict the eGFR within 7 days after PN (Table 3, Supplementary Table 4). Older age, larger tumor size, lower preoperative eGFR, higher serum cystatin C, and shorter TFS were correlated with worse short-term eGFR. The marginal and conditional *R*^2^ values were 0.656 and 0.875, respectively.

## Discussion

The prediction of postoperative eGFR can help make informed choices between RN and PN, especially for complex renal tumors. Tc-99 m DTPA RDI has been widely used to measure split RF [20]. However, how to use this important information about split rGFR to predict postoperative eGFR lacks computational models. Hence, we developed a pretreatment tool for predicting eGFR based on split rGFR.

For example, a 56-year-old man with a rGFR of 24 ml/min/1.73 m^2^ on the operative side, a rGFR of 36 ml/min/1.73 m^2^ on the healthy side, serum cystatin C of 0.91 mg/L, and a preoperative eGFR of 67 ml/min/1.73 m^2^ had a history of hypertension and diabetes. If RN was performed, the predicted eGFRs were 50, 53 and 55 ml/min/1.73 m^2^ at 2 years after open, laparoscopic and robotic surgery, respectively. If PN was performed, the predicted eGFRs were 67, 73 and 73 ml/min/1.73 m^2^ at 2 years after open, laparoscopic and robotic surgery, respectively. To avoid stage 3 chronic kidney disease, PN would be the preferred treatment, especially laparoscopic and robotic PN. In addition, because the predicted eGFRs were greater than 45 ml/min/1.73 m^2^ after RN, RN was also feasible according to American Urological Association guidelines [5]. Thus, if clinicians only focus on the preoperative rGFR of 36 ml/min/1.73 m^2^ on the healthy side, they will not be able to make an objective judgment of postoperative eGFR.

Split rGFR is at the core of the prediction models. In our study, higher rGFR of the healthy side and lower rGFR of the operative side meant higher eGFR after RN. Moreover, patients who underwent RN with higher rGFR on the healthy side also had more recovery of eGFR over time.

Previous study suggested that patients underwent laparoscopic PN maintained slightly higher eGFR than those underwent open PN, and researchers explained this phenomenon as the transient ischemia caused by pneumoperitoneum could limit renal reperfusion injury [21]. Additionally, another study suggested that robotic PN allows better preservation of split eGFR than laparoscopic PN by increasing the parenchymal preservation ratio [22]. Our models further demonstrated the differences in postoperative eGFR among these three surgical methods. For long-term eGFR after PN, laparoscopic and robotic surgery were superior to open surgery, possibly because of the above reasons. Interestingly, for long-term eGFR after RN, laparoscopic and robotic surgery were also superior to open surgery. We speculate that minimally invasive technology reduced overall trauma, which indirectly benefited the recovery of eGFR. The eGFR difference between laparoscopic RN and robotic RN decreased over time, indicating that robotic RN was more conducive to early recovery of RF. To our knowledge, few studies have reported the protective effect of minimally invasive technology on eGFR after RN. Importantly, this indicates that for patients with poor rGFR on the healthy side, if RN is chosen, minimally invasive surgery should also be given priority, as it is likely to change the outcome of renal insufficiency.

Generally, the merits of this study were clear. First, we used a large cohort of 2251 patients for long-term model and 2447 patients for short-term model. Based on this large cohort, our models can predict eGFR at many time points after surgery, not just at a single time point as in most studies. Second, we utilized the information of split rGFR, which enabled our models to have high prediction ability for the eGFRs after RN. In other prediction models not based on split RF, the prediction ability for RN was significantly lower than that for PN [11].

However, this study has several limitations. Above all, the analysis is based on retrospective data from a single center, which can be associated with information bias and selection bias. Additionally, data on tumor complexity, such as the RENAL nephrometry score [23], 24-h proteinuria [24], co-existing comorbidities other than diabetes and hypertension, lifestyle factors as smoking or alcohol use, or medication usage were not included. These parameters may improve the prediction ability, but they will also greatly increase the difficulty of clinical usage. Finally, the accuracy of split rGFR measurements by Tc-99 m DTPA RDI is different in patients with different degrees of RF impairment, which generally underestimates GFR [25]. Note that we use rGFR to predict eGFRs, the deviation between rGFR and actual single-kidney GFR does not affect the application of the models.

In conclusion, our study developed novel tools to accurately predict short-term and long-term eGFRs after RN or PN based on split rGFR, and that is helpful for preoperative decision-making and communication. A minimally invasive approach seem to improve postoperative eGFRs after both PN and RN, which needs more evidence to confirm. Although awaiting external validation, the results of this study may supplement the surgical guidelines for patients with renal tumors.

### Supplementary Information

Below is the link to the electronic supplementary material.

Supplementary file1 (DOCX 2269 KB)

## Data Availability

The datasets analyzed during this study are available from the corresponding authors upon reasonable request.
