# Supplementary material for: Predicting estimated glomerular filtration rate after partial and radical nephrectomy based on split renal function measured by radionuclide: a large-scale retrospective study
Source: World J Urol. 2023 Oct 31;41(12):3567–73. doi: 10.1007/s00345-023-04686-4 (PMC10693500; doi:10.1007/s00345-023-04686-4)
Supplement: Supplementary file 1 — Supplementary file1 (DOCX 2269 KB) [file 345_2023_4686_MOESM1_ESM.docx]

**Supplementary Material**

**Supplementary Fig. 1** Study flow chart

**Supplementary Fig. 2** Spearman’s correlation coefficients between preoperative features

**Supplementary Table 1** Results of model-simplification to predict long-term eGFR after radical nephrectomy

**Supplementary Table 2** Results of model-simplification to predict long-term eGFR after partial nephrectomy

**Supplementary Table 3** Results of model-simplification to predict short-term eGFR after radical nephrectomy

**Supplementary Table 4** Results of model-simplification to predict short-term eGFR after partial nephrectomy

**Supplementary Fig. 1 Study flow chart**


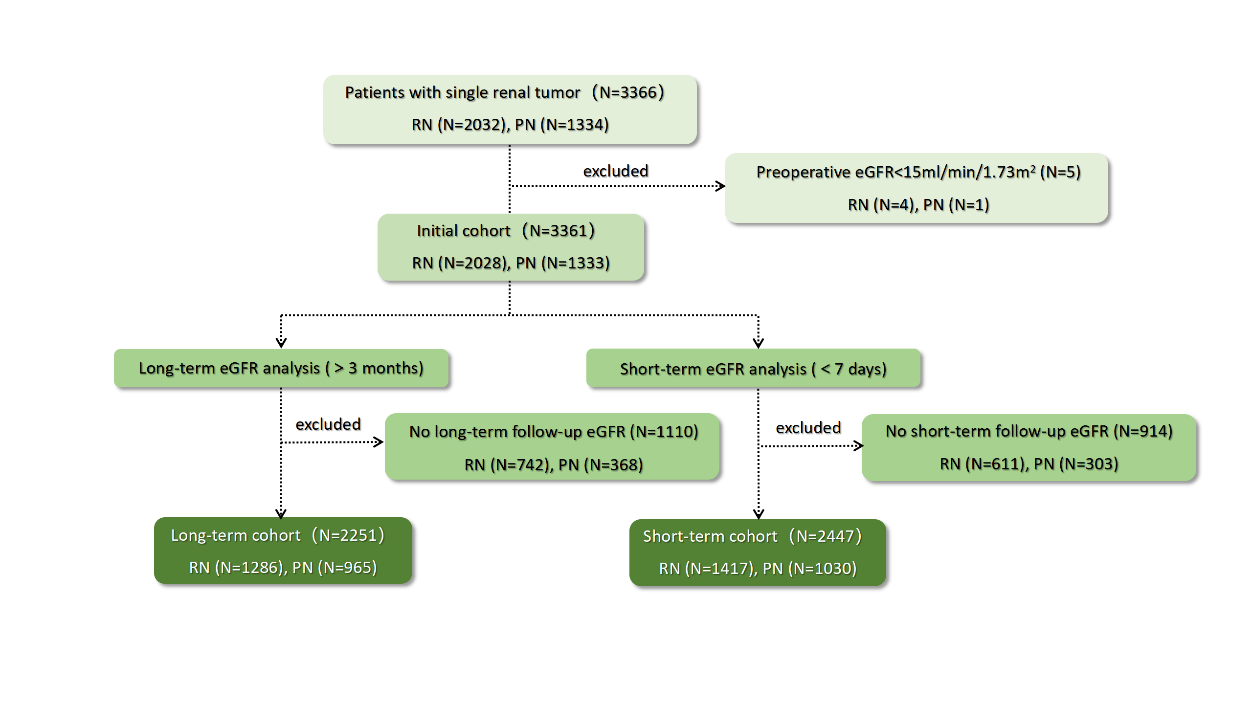


eGFR, estimated glomerular filtration rate; PN, partial nephrectomy; RN, radical nephrectomy.

**Supplementary Fig. 2 Spearman’s correlation coefficients between preoperative features**


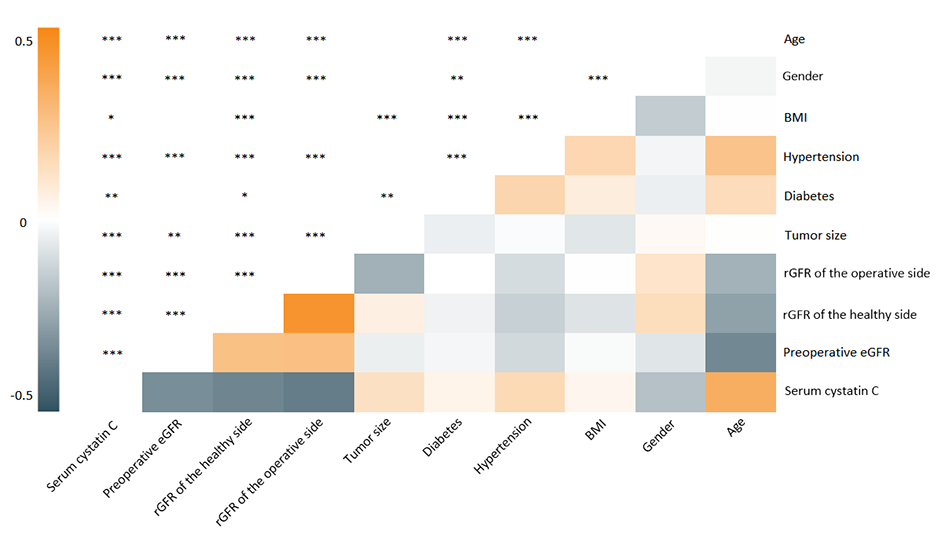


BMI, body mass index; eGFR, estimated glomerular filtration rate; rGFR, glomerular filtration rate measured by radionuclide. **P* < 0.05, ***P* < 0.01, ****P* < 0.001

**Supplementary Table 1 Results of model-simplification to predict long-term eGFR after radical nephrectomy**

| **Fixed effects** | **DF** | **AIC** | **Chi-sq** | ***p* value** |
| --- | --- | --- | --- | --- |
| **Elimination of terms, in sequence:** |  |  |  |  |
| *Full model* |  | 20358 |  |  |
| - *Age × Time* | 1 | 20356 | 0.02 | .89 |
| - *RGFR of the operative side × Time* | 1 | 20354 | 0.10 | .76 |
| - *Hypertension × Time* | 1 | 20354 | 1.84 | .17 |
| - *BMI × Time* | 1 | 20353 | 0.80 | .37 |
| - *BMI* | 1 | 20351 | <.001 | .99 |
| - *Gender* *×* *Time* | 1 | 20351 | 1.94 | .16 |
| - *Hypertension* | 1 | 20352 | 2.87 | .09 |
| - *Gender* | 1 | 20351 | 1.47 | .23 |
| - *Tumor size × Time* | 1 | 20351 | 1.76 | .18 |
| - *Diabetes × Time* | 1 | 20353 | 4.21 | .04 |
| - *Tumor size* | 1 | 20357 | 5.49 | .02 |
| **Remaining model terms:** |  |  |  |  |
| *Age* | 1 |  | 10.32 | .001 |
| *Preoperative eGFR* | 1 |  | 720.51 | <.001 |
| *RGFR of the operative side* | 1 |  | 30.45 | <.001 |
| *RGFR of the healthy side* | 1 |  | 131.35 | <.001 |
| *Cystatin C* | 1 |  | 11.56 | <.001 |
| *Diabetes* | 1 |  | 10.17 | .001 |
| *Surgical methods* | 2 |  | 14.90 | <.001 |
| *Time* | 1 |  | 389.69 | <.001 |
| *Preoperative eGFR × Time* | 1 |  | 46.98 | <.001 |
| *RGFR of the healthy side × Time* | 1 |  | 17.35 | <.001 |
| *Cystatin C × Time* | 1 |  | 30.20 | <.001 |
| *Surgical methods × Time* | 2 |  | 11.29 | .004 |

BMI, body mass index; eGFR, estimated glomerular filtration rate; rGFR, glomerular filtration rate measured by radionuclide.

Term removals are indicated by “-”.

**Supplementary Table 2 Results of model-simplification to predict long-term eGFR after partial nephrectomy**

| **Fixed effects** | **DF** | **AIC** | **Chi-sq** | ***p* value** |
| --- | --- | --- | --- | --- |
| **Elimination of terms, in sequence:** |  |  |  |  |
| *Full model* |  | 17213 |  |  |
| - *Tumor size × Time* | 1 | 17211 | 0.002 | .97 |
| - *Gender × Time* | 1 | 17209 | 0.002 | .97 |
| - *Diabetes × Time* | 1 | 17207 | 0.31 | .58 |
| - *Hypertension × Time* | 1 | 17206 | 0.32 | .57 |
| - *Diabetes* | 1 | 17204 | 0.003 | .96 |
| - *Gender* | 1 | 17202 | 0.22 | .64 |
| - *Hypertension* | 1 | 17200 | 0.29 | .59 |
| - *RGFR of the healthy side × Time* | 1 | 17199 | 0.94 | .33 |
| - *RGFR of the healthy side* | 1 | 17199 | 1.89 | .17 |
| - *Tumor size* | 1 | 17201 | 4.00 | .05 |
| - *BMI × Time* | 1 | 17204 | 5.14 | .02 |
| - *BMI* | 1 | 17204 | 1.84 | .17 |
| - *RGFR of the operative side × Time* | 1 | 17206 | 4.19 | .04 |
| - *RGFR of the operative side* | 1 | 17209 | 4.97 | .03 |
| **Remaining model terms:** |  |  |  |  |
| *Age* | 1 |  | 34.89 | <.001 |
| *Preoperative eGFR* | 1 |  | 925.17 | <.001 |
| *Cystatin C* | 1 |  | 62.25 | <.001 |
| *Surgical methods* | 2 |  | 25.62 | <.001 |
| *Time* | 1 |  | 279.24 | <.001 |
| *Age × Time* | 1 |  | 14.62 | <.001 |
| *Preoperative eGFR × Time* | 1 |  | 63.80 | <.001 |
| *Cystatin C × Time* | 1 |  | 32.86 | <.001 |
| *Surgical methods × Time* | 2 |  | 12.66 | .002 |

BMI, body mass index; eGFR, estimated glomerular filtration rate; rGFR, glomerular filtration rate measured by radionuclide.

Term removals are indicated by “-”.

**Supplementary Table 3 Results of model-simplification to predict short-term eGFR after radical nephrectomy**

| **Fixed effects** | **DF** | **AIC** | **Chi-sq** | ***p* value** |
| --- | --- | --- | --- | --- |
| **Elimination of terms, in sequence:** |  |  |  |  |
| *Full model* |  | 10532 |  |  |
| - *Diabetes × Time* | 1 | 10530 | 0.03 | .85 |
| - *Surgical methods × Time* | 2 | 10527 | 0.48 | .79 |
| - *RGFR of the operative side × Time* | 1 | 10525 | 0.15 | .70 |
| - *Gender × Time* | 1 | 10524 | 0.84 | .36 |
| - *Cystatin C × Time* | 1 | 10522 | 0.70 | .40 |
| - *Age × Time* | 1 | 10521 | 1.01 | .32 |
| - *Hypertension × Time* | 1 | 10521 | 1.94 | .16 |
| - *BMI × Time* | 1 | 10521 | 2.15 | .14 |
| - *Surgical methods* | 2 | 10519 | 1.39 | .50 |
| - *Hypertension* | 1 | 10518 | 0.80 | .37 |
| - *Gender* | 1 | 10517 | 1.43 | .23 |
| - *Diabetes* | 1 | 10517 | 1.84 | .18 |
| - *Preoperative eGFR × Time* | 1 | 10518 | 3.38 | .07 |
| - *Cystatin C* | 1 | 10520 | 3.89 | .05 |
| - *Tumor size × Time* | 1 | 10523 | 4.63 | .03 |
| - *Tumor size* | 1 | 10525 | 4.39 | .04 |
| **Remaining model terms:** |  |  |  |  |
| *Age* | 1 |  | 32.46 | <.001 |
| *BMI* | 1 |  | 36.42 | <.001 |
| *Preoperative eGFR* | 1 |  | 960.83 | <.001 |
| *RGFR of the operative side* | 1 |  | 137.02 | <.001 |
| *RGFR of the healthy side* | 1 |  | 274.11 | <.001 |
| *Time* | 1 |  | 67.01 | <.001 |
| *RGFR of the healthy side × Time* | 1 |  | 24.55 | <.001 |

BMI, body mass index; eGFR, estimated glomerular filtration rate; rGFR, glomerular filtration rate measured by radionuclide.

Term removals are indicated by “-”.

**Supplementary Table 4 Results of model-simplification to predict short-term eGFR after partial nephrectomy**

| **Fixed effects** | **DF** | **AIC** | **Chi-sq** | ***p* value** |
| --- | --- | --- | --- | --- |
| **Elimination of terms, in sequence:** |  |  |  |  |
| *Full model* |  | 9641.3 |  |  |
| - *Cystatin C × Time* | 1 | 9639.3 | 0.0007 | .98 |
| - *Hypertension × Time* | 1 | 9637.3 | 0.004 | .95 |
| - *Surgical methods × Time* | 2 | 9633.5 | 0.26 | .88 |
| - *Preoperative eGFR × Time* | 1 | 9631.5 | 0.003 | .96 |
| - *Diabetes × Time* | 1 | 9629.6 | 0.05 | .83 |
| - *RGFR of the healthy side × Time* | 1 | 9627.6 | 0.06 | .81 |
| - *Hypertension* | 1 | 9625.8 | 0.19 | .66 |
| - *Age × Time* | 1 | 9624.3 | 0.45 | .50 |
| - *RGFR of the operative side × Time* | 1 | 9622.9 | 0.64 | .42 |
| - *Tumor size × Time* | 1 | 9621.5 | 0.57 | .45 |
| - *RGFR of the operative side* | 1 | 9620.0 | 0.55 | .46 |
| - *Diabetes* | 1 | 9619.8 | 1.82 | .18 |
| - *RGFR of the healthy side* | 1 | 9620.0 | 2.21 | .14 |
| - *Gender × Time* | 1 | 9620.4 | 2.31 | .13 |
| - *Gender* | 1 | 9620.9 | 2.50 | .11 |
| - *Surgical methods* | 2 | 9621.3 | 4.40 | .11 |
| - *BMI × Time* | 1 | 9624.2 | 4.97 | .03 |
| - *BMI* | 1 | 9623.0 | 0.72 | .40 |
| **Remaining model terms:** |  |  |  |  |
| *Age* | 1 |  | 27.19 | <.001 |
| *Tumor size* | 1 |  | 24.25 | <.001 |
| *Preoperative eGFR* | 1 |  | 828.67 | <.001 |
| *Cystatin C* | 1 |  | 18.65 | <.001 |
| *Time* | 1 |  | 113.50 | <.001 |

BMI, body mass index; eGFR, estimated glomerular filtration rate; rGFR, glomerular filtration rate measured by radionuclide.

Term removals are indicated by “-”.
